# Serum SNTF, a Surrogate Marker of Axonal Injury, Is Prognostic for Lasting Brain Dysfunction in Mild TBI Treated in the Emergency Department

**DOI:** 10.3389/fneur.2020.00249

**Published:** 2020-04-08

**Authors:** Robert Siman, Hongmei Cui, Sandi S. Wewerka, Lydia Hamel, Douglas H. Smith, Michael D. Zwank

**Affiliations:** ^1^Department of Neurosurgery, Center for Brain Injury and Repair, Perelman School of Medicine, University of Pennsylvania, Philadelphia, PA, United States; ^2^Department of Emergency Medicine, Regions Hospital, St. Paul, MN, United States

**Keywords:** mild traumatic brain injury, prognostic biomarker, diffuse axonal injury, blood test, post-concussion syndrome, calpain, SNTF

## Abstract

Mild traumatic brain injury (mTBI) causes persisting post-concussion syndrome for many patients without abnormalities on conventional neuroimaging. Currently, there is no method for identifying at-risk cases at an early stage for directing concussion management and treatment. SNTF is a calpain-derived N-terminal proteolytic fragment of spectrin (α_II_-spectrin1-1176) generated in damaged axons following mTBI. Preliminary human studies suggest that elevated blood SNTF on the day of mTBI correlates with white matter disruption and lasting brain dysfunction. Here, we further evaluated serum SNTF as a prognostic marker for persistent brain dysfunction in uncomplicated mTBI patients treated in a Level I trauma center emergency department. Compared with healthy controls (*n* = 40), serum SNTF increased by 92% within 24 h of mTBI (*n* = 95; *p* < 0.0001), and as a diagnostic marker exhibited 100% specificity and 37% sensitivity (AUC = 0.87). To determine whether the subset of mTBI cases positive for SNTF preferentially developed lasting brain dysfunction, serum levels on the day of mTBI were compared with multiple measures of brain performance at 90 days post-injury. Elevated serum SNTF correlated significantly with persistent impairments in cognition and sensory-motor integration, and predicted worse performance in each test on a case by case basis (AUC = 0.68 and 0.76, respectively). SNTF also predicted poorer recovery of cognitive stress function from 30 to 90 days (AUC = 0.79–0.90). These results suggest that serum SNTF, a surrogate marker for axonal injury after mTBI, may have potential for the rapid prognosis of lasting post-concussion syndrome and impaired functional recovery following CT-negative mTBI. They provide further evidence linking axonal injury to persisting brain dysfunction after uncomplicated mTBI. A SNTF blood test, either alone or combined with other markers of axonal injury, may have important utilities for research, prognosis, management and treatment of concussion.

## Introduction

Mild traumatic brain injury (mTBI), often referred to as concussion, is the most common neurological injury, occurring at an estimated annual incidence in the United States of 1.4–3.8 million (1). For TBI sufferers with mild initial symptoms, conventional neuroimaging such as non-contrast head CT scan is usually negative, and post-concussion symptoms commonly resolve within a few hours or days. Nevertheless, in a significant subset of these uncomplicated mTBI cases, brain dysfunction is persistent and its effects debilitating, sometimes for years (2–7). Unfortunately, there is currently no therapeutic or rehabilitation intervention clinically proven to promote long-term brain functional outcomes after CT-negative mTBI. Moreover, there is no established method for identifying at an early stage those individuals at risk of experiencing lasting brain dysfunction. For mTBI management, challenges remain to make neurobiologically informed decisions on suitability for return to work, school, play, or military service, and assess the vulnerability to repetitive injuries. Progress has been made toward understanding the pathophysiology of mTBI that leads to chronic post-concussion syndrome. In particular, diffuse axonal injury (DAI) has emerged as a likely primary structural correlate for chronic brain functional impairment after mTBI. Axonal pathology is reportedly extensive in human mTBI cases who then died shortly thereafter from other causes (8). In a large animal model mimicking the human biomechanics of mTBI, DAI accompanied by localized blood-brain barrier disruption are the only observable histopathologies (9–12). From advanced neuroradiology techniques such as diffusion tensor imaging, white matter microstructural disruption is strongly expressed in some mTBI patients (13–16). A blood biomarker whose levels relate to mTBI-induced chronic brain dysfunction and the DAI underlying it could have major applications related to mTBI.

SNTF is an N-terminal proteolytic fragment of spectrin (α_II_-spectrin1-1176) that is generated by the calpain family of calcium-activated proteases under neurodegenerative conditions (17–19). SNTF is normally undetectable in neurons and their axons, but is formed within injured neurons via calcium overload and calpain-mediated spectrin degradation, after which it is released (20). SNTF preferentially accumulates within damaged axons in both small and large animal models of mTBI (21–24) as well as in human TBI cases examined post-mortem (24). In a preliminary study of mTBI patients treated in the emergency department, blood SNTF levels in the acute post-injury period are associated with white matter abnormalities detectable with diffusion tensor imaging, as well as a measure of cognitive dysfunction persisting for at least 3 months (25). Serum SNTF also increases rapidly in a subset of concussed professional ice hockey players, and distinguishes those that experience lasting symptoms requiring prolonged delay in their return to play (26). Taken together, these studies support the hypothesis that SNTF is a biologically plausible surrogate marker for DAI, and its blood elevation following mTBI provides preliminary biomarker evidence linking DAI with long-term brain functional impairment. This hypothesis is further supported by studies of additional candidate markers for mTBI. In sports-related concussion, the axon-enriched cytoskeletal proteins tau and neurofilament L (NFL) also increase in the blood acutely after injury, where their levels above threshold relate to the persistence of symptoms (27, 28) and, in the case of tau, correlate with the elevation in SNTF (26). However, studies of tau and NFL as prognostic markers for mTBI treated in the emergency department included complicated TBI cases with intracranial lesions discernable on non-contrast head CT scan, which may be prognostic for worse long-term outcomes (29). Thus far, neither marker has shown utility in this setting for the prognosis of uncomplicated mTBI (30–32). Biomarkers tied to brain elements other than the axon also have been investigated as candidates for mTBI. Peripheral blood levels of a tandem of the astrocyte-enriched glial fibrillary acidic protein (GFAP) and the neuron-enriched ubiquitin C-terminal hydrolase-L1 (UCH-L1) identify cases of complicated TBI with intracranial lesions and distinguish them with high fidelity from cases of mTBI (33–37). However, these and other well studied proteins such as S100β and neuron-specific enolase have yet to demonstrate prognostic utility in the much larger uncomplicated mTBI patient population (27, 31, 37).

Given the urgent and currently unmet need for a rapid and technically simple prognostic test for mTBI, it is important to further assess surrogate markers for DAI measurable in the blood. Here, we conducted a study evaluating serum SNTF as a rapid prognostic marker for persistent brain dysfunction in mTBI patients treated in the emergency department of a Level I trauma center. The study excluded complicated TBI, and focused on 95 mTBI participants who either were confirmed as head CT negative or did not meet criteria to receive a head CT scan. We compared the serum levels of SNTF on the day of mTBI with its levels in uninjured controls, and evaluated prognostic relationships between the day of injury serum SNTF concentrations and the presence of persistent brain dysfunction, using a battery of tests of cognitive and sensory-motor-integration performance, conducted at two time points in the chronic post-injury period.

## Methods

### Study Participants

The prospective study of mild traumatic brain injury (mTBI) treated in the emergency department was reviewed and approved by the Institutional Review Boards of Regions Hospital, St. Paul, and the University of Pennsylvania (#810115). All participants in this study provided written informed consent, or assent if written consent was given by a minor's parent. They were recruited and assessed with approval from and according to the ethical guidelines of the Institutional Review Board of Regions Hospital. All procedures were conducted in accord with the ethical standards of the Helsinki Declaration of 1975, as revised in 2000.

This study on SNTF (α_II_-spectrin1-1176) as a potential diagnostic and prognostic marker examined sera of 40 healthy control participants (20 males and 20 females) and 95 mild TBI patients (55 males and 40 females), with the latter samples collected within 24 h of injury. The time between mTBI and blood sampling ranged from 2 to 22 h, with 87% collected from 2 to 7 h post-injury. The mTBI cases are typical of those evaluated in the emergency department setting and then discharged without hospital admission. They presented with initial GCS scores of 13–15 and were recruited from a convenience sample of patients treated in the emergency department of the level I trauma center at Regions Hospital, St. Paul. The mTBI patients were > 9 years of age, had an injury to the head from blunt trauma or acceleration-deceleration forces, experienced observed or self-reported confusion, disorientation, or impaired consciousness (<5 min), and one or more of the following concussion symptoms: dizziness, headache, fatigue, irritability, vomiting. Non-contrast head CT scans were performed on 80 mTBI study participants, with the remaining 15 cases not meeting criteria to receive a head CT scan. Decisions to perform CT scan were made based on clinician experience and were frequently guided by the use of clinical decision rules (38). All of the mTBI study participants were discharged without hospital admission.

Exclusion criteria for enrollment were: age under 10 years, non-English speaking, abnormal acute intracranial CT findings, blood alcohol level >200 mg/dl, previous head injury within the past 30 days, pre-existing neurological disorder, or pre-existing psychiatric disorder. In the sections that follow herein, we refer to cases having an initial GCS of 13–15 either not meeting criteria for head CT scanning or with negative findings on a non-contrast head CT scan as mTBI. Healthy controls were free of known diseases, and no history of TBI.

### Brain Functional Assessments

After enrollment, the mTBI participants were administered the Standardized Assessment of Concussion (SAC) and the Rivermead Post-Concussion Symptom Questionnaire (RPCSQ). The RPCSQ tested 16 different self-reported symptoms and was performed by study participants on a weekly basis for up to 90 days post-injury (*n* = 58 at 90 days).

At 30 and 90 days post-injury, mTBI study participants were administered a battery of five neurocognitive performance tests: the Stroop Color and Word, Digit Span, Trail Making, Buschke Selective Reminding, and Controlled Oral Word Association test. From 65 to 67 participants completed these cognitive tests at 90 days, depending on the test. One mTBI patient, an 86 years old female, was excluded from the comparative analyses of serum SNTF levels and neurobehavioral test performance, owing to the established deleterious effects of this advanced age on performance in the battery of cognitive and sensory-motor integration tests (39–46). To assess recovery of cognitive function, 30 days scores were subtracted from 90 days scores for each concussion case in the Stroop Color and Word Interference Test (*n* = 65), measuring cognitive function under stress.

Sensory-motor integration was evaluated using the Grooved Pegboard test (*n* = 65 at 90 days). The speed to complete the test and the number of errors made were determined for both the dominant and non-dominant hands. For comparative assessment of serum SNTF levels in relation to persisting sensory-motor integration dysfunction, participants were dichotomized to either good or bad functional groups, with the cutoff criteria for the latter defined as either taking 80 s or more to complete the dominant hand test (placing them at least 20% above the mean of all mTBI patients), or committing two or more dominant hand errors. Eleven mTBI cases (17%) met these criteria at 90 days post-injury for dysfunctional sensory-motor integration.

### SNTF Second Generation Immunoassay

Development of a second-generation electrochemiluminescence-based SNTF sandwich immunoassay improved our previously published method, increasing the detection sensitivity for serum SNTF by more than an order of magnitude (25, 26), and facilitating quantification of the low SNTF levels in sera from healthy control subjects. One major change involved the derivation of an ultra-high affinity rabbit monoclonal cleavage site-specific antibody for SNTF, selected based on its superior performance as the detector in the sandwich immunoassay format. The rabbit monoclonal was derived under contract with Abcam. Rabbits were immunized with the synthetic peptide CAQQEVY, a mimic of the neoantigenic site at the carboxy-terminal region of the amino-terminal half of human α_II_-spectrin generated by calpain cleavage between residues tyrosine 1,176 and glycine 1,177 (19, 47). The peptide was conjugated via its cysteine side chain to carrier proteins keyhole limpet hemocyanin and bovine serum albumin (BSA) using maleimide chemistry, with the former conjugate used for the initial immunization and all but the final boost, and the latter for the boost prior to the hybridoma fusion. Hybridomas were screened by a two-step procedure, initially by testing conditioned media against ELISA plates coated with the peptide-BSA conjugate, and subsequently evaluating all of the initial positives as detector antibodies in electrochemiluminescence sandwich immunoassay with SNTF standard. Following subcloning and a second round of the two-step screening, the rabbit monoclonal antibody 43–8 was selected for scale-up production, and purified to >95% homogeneity using protein A affinity chromatography. Specificity of the rabbit monoclonal for SNTF was established by Western blot analysis (>1,500-fold selectivity for SNTF over intact α_II_-spectrin), and by the more than order of magnitude difference in signal in sandwich immunoassay between SNTF-enriched and SNTF-poor brain extracts.

SNTF was quantified in human sera by the following modifications of our original immunoassay method (25, 26). Mouse monoclonal anti-α_II_-spectrin capture antibody (clone D8/B7; BioLegend) was biotinylated, and bound to 96 well electrochemiluminescence plates spot coated with streptavidin (MesoScale Discovery), then the wells blocked with BSA. The mouse monoclonal reacts with the SH3 domain spanning residues 967–1,026 and recognizes SNTF as well as the intact α_II_ subunit, but not carboxy-terminal α_II_-spectrin derivatives of calpain and caspase proteolysis referred to in the literature as SBDPs 150, 145, or 120 (48), all of which lack this domain. Sera were diluted to 40%, incubated at 25 μl/well for 2 h, the wells washed, and incubated for 1 h in SNTF-specific rabbit monoclonal 43–8 at 0.1 μg/ml, and finally for 1 h in goat-anti-rabbit IgG-Sulfotag (species cross-adsorbed; MesoScale Discovery). Electrochemiluminescent signals were quantified using the QuickPlex SQ120 imaging system (MesoScale Discovery), and standardized against serial dilutions of either partially purified brain-derived SNTF (25) or recombinant human SNTF (rhSNTF). The latter was generated by digesting purified recombinant human full-length α_II_-spectrin (Origene) with purified human calpain I (Millipore-Sigma; 100:1 molar ratio). The second generation SNTF immunoassay has an experimentally determined lower limit of detection (LLOD) of 0.31 Units SNTF (1 Unit corresponds to the amount in nanoliters of SNTF-containing standard per ml), corresponding to 32 femtograms rhSNTF/well. The lower limit of quantification (LLOQ) of the second generation immunoassay is 0.55 U, or 58 femtograms rhSNTF/well. The coefficient of variation, determined using 16 human serum samples analyzed a minimum of three times, is 13.6%. Spiking experiments adding known amounts of SNTF standard to pooled healthy human control serum demonstrated equivalent and high recovery across the full range of SNTF concentrations. Systematic testing of repetitive freeze-thaw demonstrated recovery of SNTF from human serum was >95% per cycle over two cycles.

SNTF was evaluated in the human sera in a blinded experimental design without knowledge of any of the study participant data. To ensure the specificity of the method for SNTF and rule out non-specific signals derived from heterophilic substances sometimes present in human sera (49), parallel wells were reacted in the same procedure as above, except that the SNTF-specific rabbit monoclonal detector was replaced with purified normal rabbit IgG at the same concentration. This procedure abolished signal, as did replacing the α_II_-spectrin-specific capture antibody with a biotinylated mouse monoclonal of the same isotype but targeting a different protein. Human serum samples were evaluated using duplicate wells per experiment in a minimum of two independent experiments per sample. As a control for assessing cross-plate variability, a pooled serum sample from healthy controls without neurological injury was evaluated on every plate.

### Statistical Analyses

Group comparisons of mean serum SNTF levels between healthy controls and mTBI cases or dichotomized serum SNTF concentrations in relation to brain functional endpoints were evaluated by the non-parametric Mann-Whitney *U*-test. Receiver Operator Characteristics (ROC) curve analyses comparing mTBI and control participants or brain functional measures for mTBI cases on the basis of their serum SNTF levels also employed the Mann-Whitney *U*-test. The comparative analyses of serum SNTF between controls and mTBI cases as well as with measures of chronic brain functional status were conducted on the mTBI group as a whole (*n* = 95), as well as the subgroup of 80 mTBI cases confirmed to have negative non-contrast head CT scans. All of the analyses were conducted with GraphPad Prism 8.0 software.

## Results

### An Improved Second Generation SNTF Immunoassay Quantifies Serum Levels in Uninjured Controls

Prior studies in experimental animal models and relatively small numbers of cases of human TBI have provided evidence that SNTF may be a biologically plausible blood biomarker for the subset of concussions that lead to diffuse axonal injury and persisting brain dysfunction (25, 26). SNTF is the predominant amino-terminal fragment of the α-subunit of the actin-binding cytoskeletal protein non-erythroid spectrin derived from cleavage by the calcium-activated calpain protease family (17–19). It is a distinct post-translationally modified protein (α_II_-spectrin1-1176) from the carboxy-terminal spectrin breakdown products, sometimes referred to in the literature as SBDPs (17–19, 48, 50, 51). In order to compare the serum SNTF level between healthy controls and uncomplicated mTBI cases on the day of injury and assess its prognostic relationship with long-term brain functional impairment, we developed an improved second-generation electrochemiluminescence-based SNTF immunoassay with high analytical validity (see *Methods* for details). The new immunoassay reliably quantified SNTF in sera from uninjured controls, with levels of the marker in 38 of 40 control sera along with all 95 mTBI patients taken within 24 h of injury being above the lower limit of quantification of 0.55 U (58 femtograms rhSNTF), and for all 135 study participants above the lower limit of detection of 0.31 U (32 femtograms rhSNTF).

### Study Cohorts and Effects of Age and Sex on Serum SNTF

**Figure 1:**
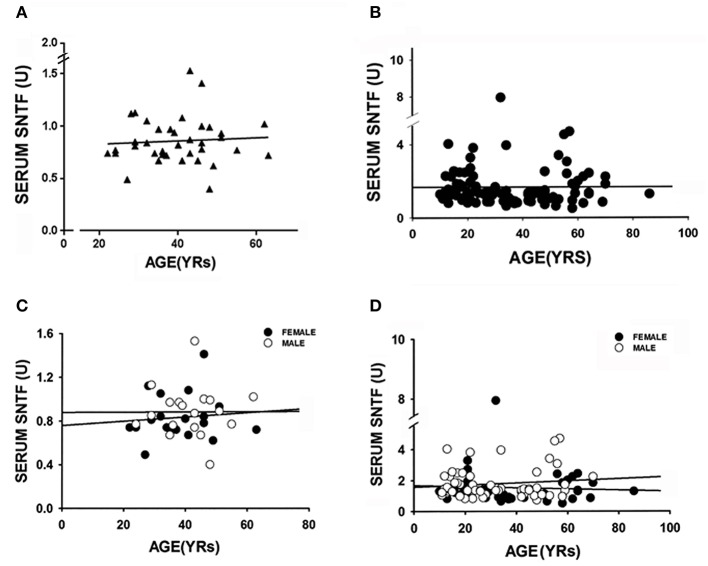
Neither age nor sex affects serum SNTF levels in either healthy controls or acutely following mild TBI. Each study participant was evaluated for serum SNTF a minimum of 4 times. Relationships between serum SNTF, age and sex were evaluated by regression analysis and Mann-Whitney *U*-test. **(A)** Serum SNTF concentration is depicted vs. the age of 40 healthy adult control subjects. Linear regression analysis shows there is no relation between serum SNTF and adult age in non-neurologic injury controls. **(B)** Serum SNTF concentration on the day of injury is depicted vs. the age of 96 mTBI study participants. Linear regression analysis shows there is no relation between serum SNTF on the day of mTBI and the age of the patient. **(C)** Serum SNTF is depicted as a function of age of healthy adult control males and females. Once again, there is no appreciable change in SNTF of either males (open circles; *n* = 20) or females (filled circles; *n* = 20) with adult aging. **(D)** Serum SNTF on the day of injury is depicted as a function of age of male (open circles; *n* = 55) and female (filled circles; *n* = 40) in the mTBI study participants. There is no appreciable change in SNTF as a function of the age of mTBI patients in either males or females.

With an objective to investigate SNTF as a prognostic blood biomarker for identifying mTBI patients at high risk of developing persisting brain dysfunction and disability, we first compared serum SNTF levels in mTBI cases on the day of injury with healthy control participants. We then analyzed SNTF levels in mTBI cases in relation to cognitive and sensory-motor test performance for up to 90 days post-injury. Table 1 shows the breakdown of the study cohorts by mean age and sex, along with the mTBI cohorts evaluated longitudinally for concussion symptoms on the day of injury, and at 30 and 90 days post-injury. Among healthy controls, serum SNTF levels were consistently low, ranging from 0.40 to 1.53 U (Figure 1A). Marker levels did not vary appreciably as a function of age (panel A, *r*^2^ = 0.01), did not differ significantly between males and females (not shown), and did not change in either males or females as a function of age (panel C; males *r*^2^ = 0.0; females *r*^2^ = 0.01). Similar observations were made in the mTBI patients, in whom serum SNTF did not differ as a function of sex (panel B; *p* > 0.5) or change appreciably with aging (panel D; males *r*^2^ = 0.01; females *r*^2^ = 0.00).

**Table 1:** Study demographics.

	**Means**			
	**N**	**Age yrs (Range)**	**Males (*N*)**	**Females (N)**
Healthy controls	40	39.3 (22–63)	40.9 ± 2.2 (20)	37.7 ± 2.3 (20)
Mild TBI				
@ < 24 h	95	35.6 (10–86)	31.9 ± 2.3 (55)	40.5 ± 3.0 (40)
@ 1 month	75	36.1 (11–70)	32.5 ± 2.5 (45)	41.6 ± 3.7 (30)
@ 3 months	65	36.4 (11–70)	35.4 ± 2.9 (39)	38.0 ± 3.7 (26)
Mild TBI/CT-				
@ 3 months	53	37.4 (11–70)	35.0 ± 3.0 (33)	41.4 ± 4.4 (20)

*The N per mild TBI group represents the number of participants that performed all of the cognitive and sensory-motor performance tests at the given time point post-injury*.

### Diagnostic Accuracy of Serum SNTF on the Day of mTBI

**Figure 2:**
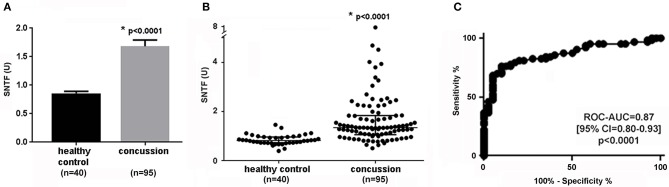
Serum SNTF is significantly elevated on the day of mTBI and is diagnostic for a subset of the injuries. Between group comparisons and ROC-AUC analyses were evaluated by Mann-Whitney *U* test. Each study participant was evaluated for serum SNTF a minimum of 4 times. **(A)** In group analysis, mean serum SNTF levels on the day of mTBI are 92% higher than their mean levels in healthy controls. The bars represent the S.E.M. Between group statistical comparison by two-tailed *t*-test. **(B)** In group analysis, median serum SNTF levels on the day of mTBI are 63% higher than their median levels in healthy controls. The filled circles represent each case, and the bars represent quartiles. Note that whereas some of the mTBI cases have serum SNTF levels indistinguishable from the range of the controls, a subset have SNTF levels markedly elevated over the entire control group. Between group statistical comparison by Mann-Whitney *U*-test. **(C)** In ROC-AUC curve analysis, serum SNTF levels on the day of mTBI distinguish a subset of injured patients from healthy controls. At a cutoff value of 1.54 U, serum SNTF is 100% specific and 37% sensitive for mTBI. The AUC is 0.87, the 95% confidence interval is 0.80–0.93, and is significantly different from chance (*p* < 0.0001; Mann-Whitney *U* test).

Compared to its level in the serum of neurologically normal uninjured controls, serum SNTF increased substantially on the day of mTBI. As shown in Figure 2, mean serum SNTF levels within 24 h of injury were 92% higher (panel A; *p* < 0.0001) and median levels were 63% higher (panel B; *p* < 0.0001). Strikingly, whereas many of the mTBI cases had serum SNTF levels indistinguishable from healthy controls, a subset had levels above those observed in all 40 controls, and up to 9-fold higher than the mean control value. Receiver Operator Characteristics-Area Under The Curve (ROC-AUC) analysis demonstrated that the level of serum SNTF on the day of injury strongly distinguished mTBI cases from uninjured controls (panel C; AUC = 0.87, *p* < 0.0001). At a cutoff value of 1.54 U, serum SNTF was 100% specific and 37% sensitive for the diagnosis of mTBI (i.e., 40 of 40 healthy controls were SNTF negative, whereas 35 of 95 mTBI cases were SNTF positive). At a lower cutoff of 1.45 U, serum SNTF retained 97.5% specificity and exhibited 40% sensitivity. The latter cutoff value was used for all of the group analyses that follow, whereas comparative evaluations between long-term brain functional performance tests and serum SNTF on a per case basis were made by ROC-AUC analyses without a pre-determined biomarker cutoff.

In subgroup analysis, serum SNTF increased on the day of mTBI in both the confirmed head CT-negative (1.72 U ± 0.12, *n* = 80) and non-scanned (1.50 U ± 0.23, *n* = 15) cases compared to healthy controls (0.86 U ± 0.03, *n* = 40; *p* < 0.0001 for both comparisons). In contrast, there was no significant difference in SNTF levels between the mTBI cases not referred for head CT and confirmed as head CT negative (*p* > 0.5). In ROC-AUC analysis comparing the head CT-negative mTBI subgroup with controls, serum SNTF on the day of injury once again strongly distinguished the two groups (AUC = 0.87; *p* < 0.0001) in a manner indistinguishable from the overall mTBI group. In this study, blood was obtained from 2 to 7 h post-injury in 88% of the mTBI cases, and there was no discernable relationship over this time frame between serum SNTF levels and the delay between injury and sampling (*r*^2^ = 0.02; *p* = 0.3).

### Evidence That Serum SNTF on the Day of mTBI Prognoses Lasting Cognitive Dysfunction and Poor Cognitive Functional Recovery

Given that in a subset of uncomplicated mTBI cases serum SNTF concentration is elevated on the day of injury above the range of neurologically normal controls, and only some mTBI cases experience discernable long-lasting brain functional problems, the question arises whether the rapid injury-induced SNTF increase relates to impaired brain performance in the chronic post-injury period. To address this issue, we compared SNTF levels on the day of mTBI with the scores from a battery of tests evaluating cognitive and sensory-motor function at 30 and 90 days post-injury. Additionally, the Standardized Assessment of Concussion served as a measure of the initial severity of symptoms on the day of injury, and the Rivermead Post-Concussion Symptom Questionnaire (RPCSQ) evaluated self-reported cognitive, somatic, and emotional symptoms throughout the 90 days observation period. Cognitive function in the chronic post-injury period was studied by serial assessments using the Stroop Color and Word, Digit Span, Trail Making, Buschke Selective Reminding, and Controlled Oral Word Association tests.

As shown by group analyses (Tables 2, 3), the serum SNTF concentration sampled within 24 h of mTBI correlated significantly with a subset of cognitive functional measures at 90 days after the injury. In the Stroop Color and Word test (Table 2), the SNTF-positive mTBI group scored poorer on the cognitive interference, color score, and color score *T* test components, with the biomarker positive mTBI group exhibiting significantly impaired performance compared with the SNTF-negative mTBI group on each of these measures. In ROC-AUC analysis, serum SNTF positivity on the day of injury significantly distinguished cases with the worst 20% of cognitive interference scores at 90 days (<49 words) from those with better test performance (>49 words; AUC = 0.68; *p* = 0.028; data not shown). In subgroup analysis of the mTBI cases with confirmed negative head CT findings, participants positive for serum SNTF on the day of injury also performed significantly worse on the Stroop cognitive interference and color score *T* test components than their SNTF-negative mTBI counterparts (Table 2).

**Table 2:** Serum SNTF on the day of mild TBI relates to cognitive and sensory-motor performance scores at 3 months post-injury.

**Performance test**	**SNTF- (*N*)**	**SNTF± (*N*)**	***P-*value**
**Stroop color and word**			
**SNTF** **>** **1.45 U:**			
Interference Score-T (words)	56.9 ± 1.5 (36)	51.5 ± 1.3 (30)	**0.015**
Color Score	75.7 ± 2.6	68.3 ± 2.1	**0.031**
Color Score-T	49.0 ± 2.2	43.3 ± 1.8	**0.050**
CT-/Interference Score-T	57.5 ± 1.9 (27)	51.8 ± 1.5 (26)	**0.040**
CT-/Color Score	74.8 ± 2.8	68.3 ± 2.3	0.068
CT-/Color Score-T	48.6 ± 2.4	42.5 ± 2.0	**0.046**
Dominant hand speed (sec)	61.4 ± 1.6 (35)	71.0 ± 4.1 (30)	0.12
Dom hand speed (>16 yrs)	60.1 ± 1.6 (30)	72.8 ± 4.8 (25)	**0.033**
Dominant hand errors	0.26 ± 0.07	0.80 ± 0.22	**0.040**
Non-Dominant hand speed	67.6 ± 2.1	71.9 ± 2.7	0.21
Non-Dominant hand errors	0.54 ± 0.15	0.70 ± 0.17	0.44
	**Mean SNTF (U)**		
	**<80 s (*****N*****)**	**≥80 s (*****N*****)**	***P-*****value**
Dominant hand speed	1.71 ± 0.14 (57)	2.58 ± 0.46 (8)	**0.019**

*The group analyses of cognitive and sensory-motor integration performance at 90 days post-injury are compared between all SNTF-negative and SNTF-positive mTBI cases, and again for cases confirmed as head CT-negative, using a cutoff value for serum SNTF concentration on the day of injury (1.445 U) that is 97.5% specific for mTBI. Cognitive performance scores were worse for both the overall mTBI SNTF-positive subgroup and for the SNTF-positive subgroup confirmed as CT negative compared to their SNTF-negative counterparts. For the sensory-motor pegboard test, mTBI cases SNTF-positive on the day of injury made significantly more dominant hand errors, and the adult mTBI subgroup also had significantly worse dominant hand speed. Serum SNTF levels were quantified a minimum of four times for each study participant. Statistically significant differences by Mann-Whitney U-test are shown in bold*.

**Table 3:** Serum SNTF on the day of mild TBI in relation to other brain performance scores at 3 months post-injury.

**Performance test**	**SNTF- (sem; *n*)**	**SNTF ± (sem; *n*)**	***P-*value**
**Rivermead post-concussion symptom questionnaire**			
SNTF > 1.45 U COG-3	1.8 ± 0.6 (32)	2.6 ± 0.6 (26)	0.38
RPQ-13	7.1 ± 1.9 (32)	8.7 ± 2.0 (26)	>0.5
**Standardized assessment of concussion**			
SNTF > 1.45 U	24.7 ± 0.4 (61)	24.9 ± 0.5 (35)	>0.5
**Digit span**			
SNTF > 1.45 U	18.0 ± 0.8 (36)	16.4 ± 0.8 (29)	0.15
**Controlled oral word association**			
SNTF > 1.45 U	39.3 ± 2.1 (37)	38.9 ± 2.4 (30)	>0.5
**Buschke selective reminding**			
SNTF > 1.45 U Reminding	54.6 ± 1.3 (37)	53.6 ± 1.4 (30)	>0.5
SNTF > 1.45 U Delayed	8.73 +/0.41 (37)	9.31 ± 0.37 (30)	0.26
**Trail making**			
SNTF > 1.45 U; Trail A speed (s)	22.5 ± 2.2 (35)	21.9 ± 1.5 (29)	>0.5
SNTF > 1.45 U; Trail B speed	52.3 ± 3.7 (35)	62.6 ± 6.7 (29)	0.16
SNTF > 1.45 U; Trail A errors	0.53 ± 0.13 (36)	0.55 ± 0.15 (29)	>0.5
SNTF > 1.45 U; Trail B errors	0.94 ± 0.45 (36)	1.28 ± 0.44 (29)	0.47

*In contrast to the significant performance impairments of SNTF-positive participants compared to the SNTF-negative cases in the test results depicted in Table 2, for other tests of cognitive function and self-reported symptomatic scores there was no significant difference as a function of serum SNTF status. The Rivermead, Digit Span, Controlled Oral Word Association, Bushke Selective Reminding, and Trail Making test data shown are at 90 days post-injury. The Standardized Assessment of Concussion was conducted on the day of injury. Serum SNTF was quantified a minimum of 4 times per mTBI study participant*.

**Figure 3:**
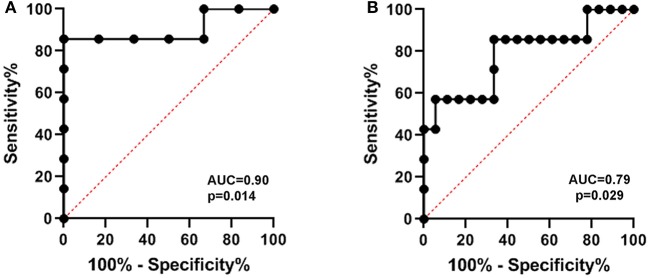
Serum SNTF on the day of mTBI predicts impaired long-term recovery of cognitive function under stress. Serum levels of the biomarker were evaluated a minimum of 4 times, and analyzed in relation to brain functional measures by Mann-Whitney *U* test. Recovery of cognitive stress function was determined as the difference in the Stroop Color and Word IS-T scores between 90 and 30 days post-injury. In ROC-AUC analyses, serum SNTF on the day of injury was significantly higher in mTBI with the decile exhibiting the worst functional recovery compared to either the best recovering decile **(A)** or best recovering quartile **(B)**.

Serum SNTF on the day of injury predicted not only impaired cognitive performance at 90 days post-injury, but also poor recovery of cognitive function under stress. Taking the difference in the Stroop interference test scores between 30 and 90 days as a measure of cognitive recovery, 41 of 65 mTBI participants improved performance over the 60 days recovery assessment period, whereas 24 showed either no improvement or worse scores. The decile showing the worst recovery of cognitive stress function had a mean SNTF level (2.57 ± 0.44) on the day of injury significantly higher than the quartile exhibiting the best cognitive recovery (1.48 ± 0.13 U; *p* = 0.004). Additional differences were observed in the median SNTF level between the worst recovering decile and the best recovering quartile (2.43 vs. 1.31 U; *p* = 0.03). As shown in Figure 3, ROC-AUC analysis demonstrated that serum SNTF on the day of injury predicted with significance mTBI cases falling into the worst recovering decile compared with both the best recovering decile (Figure 3A; AUC = 0.90, *p* = 0.014) and the best recovering quartile (Figure 3B; AUC = 0.79; *p* = 0.029).

One trivial possibility that could account for differences in long-term cognitive performance between the SNTF-positive and –negative mTBI groups is potential imbalance between the two in the initial severity of the mTBI. However, as shown in Table 3, scores on the Standardized Assessment of Concussion conducted on the day of injury demonstrated that the differences in long-term cognitive performance and functional recovery in relation to acute serum SNTF levels were not due to any appreciable difference in the initial severity of the concussion symptoms between the serum SNTF-positive and -negative groups.

The relationships in mTBI cases between serum SNTF concentrations and cognitive functional test scores in the chronic post-injury period and were subtle. Unlike for multiple readouts for the Stroop cognitive test, four other assessments of cognition at 90 days after mTBI did not reveal significant performance differences in association with the day of injury serum SNTF levels. Scores on the Digit Span, Trail Making, Buschke Selective Reminding, and Controlled Oral Word Association tests did not differ with statistical significance between the SNTF-negative and –positive mTBI groups, although the SNTF-positive mTBI participants trended toward worse performance in the Digit Span, Trail Making B, and Buschke Delayed Reminding tests (Table 3). In addition, the Rivermead Post-Concussion Symptom Questionnaire, a self-reporting tool for assessing cognitive problems via the COG-3 sub-score, or emotional and somatic symptoms via the RPQ-13 sub-score, did not reveal self-reported symptomatic differences that associated with the day of injury serum SNTF concentration.

### Evidence that Serum SNTF on the Day of mTBI Is Prognostic for Persistent Impairment in Sensory-Motor Integration

In addition to the evidence associating serum SNTF positivity on the day of injury with long-lasting impairment in cognition measured using the Stroop Color and Word test, the SNTF-positive mTBI patients also exhibited worse sensory-motor integration function at 90 days post-injury, as assessed using the Grooved Pegboard test. As shown in Table 2, the SNTF-positive mTBI group completed the task with a slower dominant hand speed that trended toward significance, and made significantly more dominant hand errors (*p* = 0.04). The association between serum SNTF on the day of mTBI and 90 day sensory-motor integration performance was stronger for the adult subgroup of mTBI cases. For mTBI participants over the age of 16, the SNTF-positive cases exhibited significantly slower dominant hand speed (72.8 s ± 4.8; *n* = 25; *p* = 0.033) compared to the SNTF-negative subgroup (60.1 s ± 1.6; *n* = 30). The SNTF-positive mTBI group also exhibited slower speed and made more errors with the non-dominant hand, although these trends were not statistically significant.

**Figure 4:**
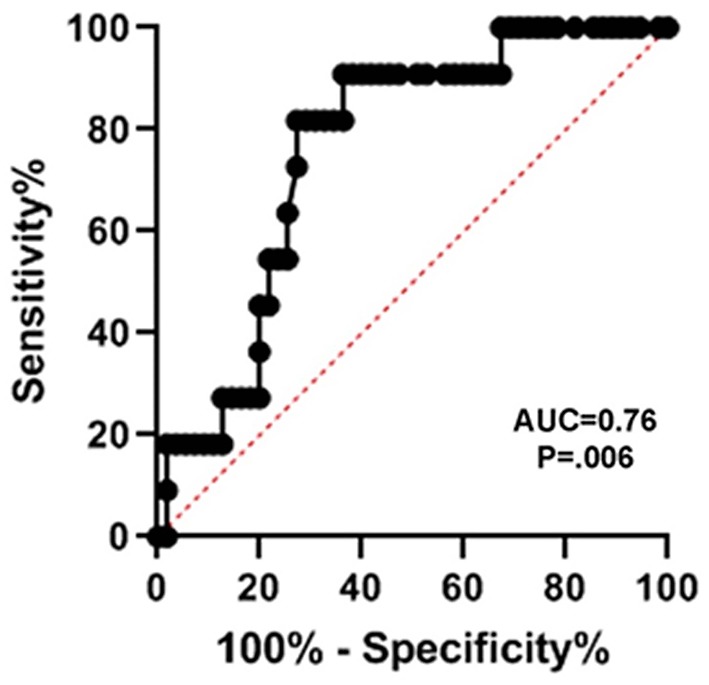
Serum SNTF on the day of mTBI predicts persisting dysfunction in sensory-motor integration at 90 days post-injury. Serum SNTF levels were analyzed for each participant a minimum of 4 times, and evaluated in relation to sensory-motor performance scores by Mann-Whitney *U* test. Sensory-motor integration function was assessed at 90 days post-injury by the Grooved Pegboard test (*n* = 65). Criteria for poor test performance were either a slow dominant hand speed to complete the test of >80 s or commission of two or more dominant hand errors. Seventeen percent of the mTBI cases met these criteria for dysfunctional sensory-motor integration at 90 days. In ROC-AUC analyses, SNTF distinguished cases with impaired sensory-motor integration at 90 days from those not meeting criteria for long-lasting dysfunction (AUC = 0.76, *p* = 0.006).

To evaluate the prognostic strength with which serum SNTF on the day of injury predicted persisting dysfunction in sensory-motor integration, ROC-AUC analysis compared performance groups based on criteria for poor functional cases that reached threshold for either slow dominant hand speed (>80 s) or dominant hand errors (2 or more; Figure 4). Eleven of the 65 cases of mTBI cases (17%) met these criteria for persisting impairment in sensory-motor integration. The SNTF cutoff of 1.45 U predicted sensory-motor dysfunction at 90 days post-injury with an AUC = 0.76 (*p* = 0.006), a sensitivity of 91% and a specificity of 64%.

## Discussion

SNTF has been reported to increase in the blood in a subset of uncomplicated mTBI patients treated in the emergency department and after sports-related concussion treated in the athletic training room, but until now has been evaluated in only relatively small numbers of cases. In the present study of 95 mTBI patients treated in a Level I trauma center emergency department setting for whom non-contrast head CT scans were either negative (*n* = 80) or not indicated (*n* = 15), serum levels of SNTF within 24 h of injury are significantly higher by 92% compared with a group of 40 neurologically normal controls matched for age and gender. A serum SNTF cutoff yielding 97.5% specificity (i.e., 39 of 40 controls were SNTF-negative) identifies 40% of mTBI cases as SNTF-positive (Figure 2; AUC = 0.87; *p* < 0.001). By comparing the functional status of SNTF-positive and -negative mTBI patients, the subset with elevated SNTF on the day of injury exhibits evidence of brain dysfunction on multiple performance tests that persist for at least 90 days post-injury. In group analyses, dichotomized SNTF discriminates lasting dysfunction in multiple measures of cognition (Table 2). It also predicts poor recovery of cognitive stress function from 30 to 90 days post-injury, separating the worst recovering decile from the best recovering quartile and decile, with AUCs of 0.79 (*p* = 0.029) and 0.90 (*p* = 0.014), respectively (Figure 3). Serum SNTF on the day of injury separates mTBI patients not only on the basis of their chronic cognitive function in the Stroop Color and Word test, but also relates strongly to persistent dysfunction in sensory-motor integration. At 90 days post-injury, the SNTF-positive mTBI group is slower to perform the Grooved Pegboard test with their dominant hand and commits significantly more errors (*p* = 0.04) than the SNTF-negative mTBI group. Significant associations with dominant hand speed and errors are even more pronounced for the adult subgroup of SNTF-positive mTBI cases over age 16. On a case-by-case basis, serum SNTF levels on the day of injury predict sensory-motor integration performance deficits at 90 days post-injury in the mTBI group overall with an AUC of 0.76 (*p* = 0.006), 91% sensitivity and 64% specificity (Figure 4). In contrast, serum SNTF does not vary appreciably as a function of age or sex in either healthy controls or on the day of injury in mTBI patients (Figure 1). These results coupled with the evidence from cognitive test performance suggest that a serum SNTF blood test on the day of injury might have important applications for rapid prognosis of mTBI.

The findings reported here from the study of 135 participants confirm and extend previous preliminary studies on small numbers of cases of SNTF as a blood biomarker for mTBI treated in the emergency department and for sports-related concussion treated in the athletic trainer's room (25, 26). Here, the development and analytical validation of a next generation SNTF electrochemiluminescence immunoassay with markedly improved detection sensitivity enables quantification of serum SNTF in healthy control as well as mTBI subjects, with all 135 participants above the lower limit of detection. There is abundant evidence that the SNTF elevation found in the blood in a subset of mTBI patients on the day of their injury derives in large part from its formation in damaged axons in the brain, leakage into the interstitial fluid, and efflux into the blood. It is a calpain-derived fragment of the α_II_-subunit of spectrin (17, 19), a large tetrameric cytoskeletal protein that binds to rings of actin filaments and links them to the inner face of the axolemma, spacing them at regular intervals (52). SNTF is not present in detectible amounts in the brain under normal conditions, but forms specifically in response to intra-axonal calcium overload, accumulates within damaged axons (18, 19), and serves as a marker for axonal cytoskeletal disruption in numerous studies of small and large animals models of mTBI, as well as in the human brain (21–24). Supporting this hypothesis, the rise in blood SNTF concentration on the day of mTBI correlates with white matter tract microstructural changes discernable by diffusion tensor imaging at 2 weeks post-injury (25). Furthermore, after concussion in professional ice hockey players, serum SNTF levels correlate with those of tau, another axon-enriched cytoskeletal protein (26). A host of histopathological and diffusion tensor imaging studies provide strong evidence that diffuse axonal injury is a structural correlate for chronic brain dysfunction following mTBI (8, 13–16). Consequently, we propose that SNTF is a biologically plausible surrogate blood biomarker for the axonal damage contributing to persisting brain functional impairments and post-concussion syndrome after mTBI.

At present there are no blood biomarkers or advanced neuroimaging methods proven and clinically validated for the rapid prognosis of mTBI. The biomarker tandem of glial fibrillry acidic protein and ubiquitin C-terminal hydrolase-L1 has consistently demonstrated utility as a blood test for identifying cases of complicated TBI presenting with mild initial symptoms (GCS 13–15) that exhibit intracranial lesions on non-contrast head CT scan (33–36). Unfortunately, in cases of mTBI with negative head CT findings, which is a far more common neurological injury, these markers have not shown prognostic utility (31, 37). In studies of sports-related mTBI evaluating concussed professional ice hockey players and boxers after a bout, tau and neurofilament L are elevated in the blood, and in the former group and in a manner similar to SNTF, predict the persistence of post-concussion symptoms based on the length of the delay in return to play. On the other hand, studies published to date of tau and NFL as candidate markers for TBI treated in the emergency department have included cases with discernable intracranial lesions on head CT scan, thus precluding evaluation of these markers for prognosis of mTBI (30–32). Strikingly, SNTF, tau, and neurofilament L are all important structural proteins of the axon whose blood levels rise after sports-related concussion (26–28), supporting the hypothesis that they all represent surrogate blood biomarkers for the diffuse axonal injury underlying functionally deleterious mTBI. This hypothesis will require further evaluation, as will the prospect for improved mTBI prognosis using a multivariable panel of biologically plausible markers.

There is confusion in the literature regarding the identity of SNTF and its characterization as a biomarker for mTBI and other acute brain injuries, owing to inconsistencies with nomenclature and the frequent lack of distinction from other calpain-derived proteolytic fragments of the α_II_-subunit of spectrin. The discovery that non-erythroid spectrin is a preferred high affinity calpain substrate came with the identification of major breakdown products of the α_II_-subunit, referred to initially as spectrin BDPs (17). Sequencing of the BDPs and development of cleavage site-specific antibodies identified the preferred calpain cleavage sites in the subunit (19, 49, 50), thereby defining the amino acid sequences of each fragment. Subsequent publications referred to the two major carboxy-terminal α_II_-spectrin fragments as SBDP150 and 145, and did not distinguish SNTF from these SBDPs (53–56). Whereas the SBDP carboxy-terminal fragments have also been studied as candidate cerebrospinal fluid and blood biomarkers for acute brain injuries, neither has shown promise as a blood diagnostic or prognostic for mTBI. In contrast, SNTF is the predominant amino-terminal calpain derivative and has been widely studied as a histological and biochemical marker for axonal degeneration and the necrotic mode of neuronal death (19, 21, 57–60). Since SNTF continues to be confused with the SBDPs in the literature, we propose for purposes of clarity adoption of the following nomenclature for calpain-derived α_II_-spectrin breakdown products: SNTF for representing spectrin α_II_ 1-1176, SBDP150 for representing α_II_ 1177-2472, and SBDP145 for representing α_II_ 1231-2472.

The present study of SNTF as a diagnostic and prognostic blood marker for lasting functional impairment after mTBI has several limitations. Advanced neuroimaging is not available from this study for examining directly the interrelationships between rapid elevations in serum SNTF, persisting brain functional problems, and white matter microstructural disruption. The association between serum SNTF elevations and the lasting effects of mTBI on cognitive performance is complex, with many tests failing to uncover any significant prognostic relationship. This issue is complicated by the well-established influences of subject intelligence level and degree of education on performance of a number of cognitive tests (39–46, 61–67). When applying tests in which performance varies widely across cases independently of mTBI, it can be challenging to discern the long-lasting effects of the injury itself. In this regard, multiple components of the Stroop Color and Word test that associate with a rapid serum SNTF increase, the Symbol Digit Modalities Test linked to blood SNTF levels previously (25), accounting for age as a covariate in cognitive testing, and the serial evaluation of these tests over a prolonged recovery time may offer advantages for future clinical assessments of long-term cognitive dysfunction and impaired functional recovery after mTBI. In addition to cognitive assessment, lasting impairments in sensory-motor integration after mTBI may be discernable using the Grooved Pegboard test. Finally, at present this study lacks comparative information on tau, neurofilament L, and other potential biomarkers for diffuse axonal injury that may have strengths when combined with SNTF in multivariable biomarker analyses. Future work will be required to address these outstanding issues and conduct a large multi-site clinical validation study of an SNTF-inclusive panel of axonal injury biomarkers.

A clinically validated prognostic blood test for mTBI tied to the pathophysiology that underlies chronic brain functional impairments would have a number of vitally important uses in precision medicine and clinical research. These include the ability to (i) give patient prognosis, facilitating neurobiologically informed decisions on return to work, school, or participation in sports or military activities; (ii) initiate rehabilitation protocols more quickly after injury; (iii) stratify mTBI to enrich for at-risk cases for participation in well-controlled clinical research studies of the underlying mechanisms and the therapeutic benefit of drug interventions and rehabilitation protocols; (iv) apply a surrogate marker for the rapid quantitative evaluation of target mechanism engagement and therapeutic benefit; and (v) encourage mTBI evaluation and treatment in the emergency department, to reduce the problem of concussion under-reporting. In summary, the results of this study of 135 participants show that, in an emergency department setting, the blood level of SNTF on the day of a CT-negative mTBI strongly discriminates a subset of injury cases and is prognostic for persistent impairments in cognitive and sensory-motor function. They provide further evidence for the important contribution of axonal injury to persisting brain dysfunction after mTBI, and suggest a day of injury SNTF blood test, either alone or combined with other markers of axonal injury, may have utility in the rapid prognosis of chronic brain dysfunction and impaired functional recovery.

## Data Availability Statement

The datasets generated for this study are available on request to the corresponding author.

## Ethics Statement

The studies involving human participants were reviewed and approved by Institutional Review Board, Regions Hospital, St. Paul, MN and Institutional Review Board, University of Pennsylvania, Philadelphia, PA. Written informed consent to participate in this study was provided by the participants' legal guardian/next of kin.

## Author Contributions

This project was conceived and developed by RS, SW, and MZ. SW and LH directed participant enrollment and were responsible for serial neurobehavioral assessments and compilation of the brain functional data sets. HC and RS performed quantitative and biostatistical analyses of serum SNTF levels comparing healthy control and mTBI participants and relating their levels to neurobehavioral outcomes in the long-term post-injury period. RS and MZ wrote the manuscript. DS supported the development of the second-generation biomarker immunoassay.

### Conflict of Interest

RS was the inventor on issued patents and pending patent applications related to an SNTF blood test for the diagnosis and prognosis of concussion. The patents are assigned to the Trustees of the University of Pennsylvania. DS was a consultant for Abbott Laboratories. The remaining authors declare that the research was conducted in the absence of any commercial or financial relationships that could be construed as a potential conflict of interest.
